# An open source pharma roadmap

**DOI:** 10.1371/journal.pmed.1002276

**Published:** 2017-04-18

**Authors:** Manica Balasegaram, Peter Kolb, John McKew, Jaykumar Menon, Piero Olliaro, Tomasz Sablinski, Zakir Thomas, Matthew H. Todd, Els Torreele, John Wilbanks

**Affiliations:** 1 Médecins Sans Frontières, Geneva, Switzerland; 2 Department of Pharmaceutical Chemistry, Philipps-University Marburg, Marburg, Germany; 3 National Institutes of Health, National Center for Advancing Translational Sciences (NCATS), Bethesda, Maryland, United States of America; 4 McGill University Institute for the Study of International Development, Montreal, Canada; 5 UNICEF/UNDP/World Bank/WHO Special Programme for Research and Training in Tropical Diseases (TDR), World Health Organization, Geneva, Switzerland; 6 Centre for Tropical Medicine and Global Health, Nuffield Department of Clinical Medicine, University of Oxford, Oxford, United Kingdom; 7 Transparency Life Sciences, New York, New York, United States of America; 8 Open Source Drug Discovery, Council of Scientific and Industrial Research (CSIR), New Delhi, India; 9 School of Chemistry, The University of Sydney, Sydney, Australia; 10 Public Health Program, Open Society Foundations, New York, New York, United States of America; 11 Sage Bionetworks, Seattle, Washington, United States of America

## Abstract

In an Essay, Matthew Todd and colleagues discuss an open source approach to drug development.

**Summary points**

- This Essay outlines how open source methods of working could be applied to the discovery and development of new medicines.
- There are many potential advantages of an open source approach, such as improved efficiency, the quality and relevance of the research, and wider participation by the scientific and patient communities; a blend of traditional and innovative financing mechanisms will have to be adopted.
- To evaluate properly the effectiveness of an open source methodology and its potential as an alternative model of drug discovery and development, we recommend that new projects be trialed and existing projects scaled up.

## Where we stand

The scientific and medical community has discovered and developed many groundbreaking medicines that have had a major impact on public health. However, drug development is challenged by a widening gap between health needs and the pharmaceutical industry’s motives and business model, alongside a decrease in efficiency per research dollar spent in medicinal product research and development (R&D), a trend known colloquially as Eroom’s Law [1].

Such fundamental challenges result in frequent high-level calls for new initiatives to develop therapeutics and bring them to market [2,3]. These include market push and pull mechanisms such as priority review vouchers, advance market commitments, and public R&D funding [4]. New organizational models have also emerged, including public–private partnerships (PPPs) [5] and not-for-profit product development partnerships (PDPs) (for example, the Drugs for Neglected Diseases Initiative [DNDi], the Medicines for Malaria Venture [MMV], and the Global Alliance for Tuberculosis Drug Development [TB Alliance]) that often apply a full “de-linkage” model in which the price of medicines and the cost of R&D are uncoupled [6,7].

## Open source pharma

An approach that has been suggested [8] but not yet fully evaluated is open source pharma. The term “open source” (OS) refers to radically transparent working practices pioneered in software development, such as the prepublication sharing of data and ideas, the possibility of participation in a project by anyone in real time, and a form of shared ownership that ensures that the underlying methods and data are public domain. In OS pharma, such principles would be applied from the discovery of a potential medicine through to its entry into the market; the impact of this, and the ease with which such principles can be applied, will vary at each stage of the process.

OS must be distinguished from “open innovation” (OI), a term used widely in the pharmaceutical industry [9]. Both are characterized by a flow of ideas between problem owners and solvers, and both therefore harness the potential of unexpected contributions. In a typical OI initiative, it is the research problem that is public domain, while research solutions are subject to the usual structures of walled competition. In OS, the problem and all potential solutions are public domain.

OS brings the greatest possible community involvement, but the transparency brings economic uncertainty in how to exploit the solutions. The underlying financial and legal structures of the pharmaceutical industry are designed for use by profit-making entities in ways that rely on monopolies, usually through patents. Under the current model, it is assumed that without exclusive ownership, there can be no guarantee of profit to shareholders—a disincentive for private sector investment in certain areas of health R&D, despite significant public investments. The requirement for a financial return causes the current system to follow priorities that can be misaligned with the greatest public health needs. The requirement for secrecy inevitably reduces the efficiency of the scientific research. The transparency at the heart of OS is thus fundamentally incompatible with traditional approaches to drug discovery and development. The involvement of the pharma industry in consortia that address neglected or tropical diseases (e.g., the Tuberculosis Drug Accelerator, which is invitation only and has no commitment to open data) ensures that a similar walled approach is usually taken even for diseases traditionally considered to yield a low return on investment.

Thus, while OS working practices are well established in some areas of software development and have a growing impact in increasing the efficiency of basic science, they have not yet been broadly applied in medical product R&D, either in academia or industry [10]. It is tempting to ascribe this to an obvious distinction: software centers on a nonphysical entity (code) that may be easily copied. Drug R&D involves tangible, lengthy, and often expensive processes. Yet, this is to obfuscate the more important point: OS is a core set of principles (defining a way of working) that can be applied quite broadly.

We propose that OS methods are a promising, yet largely untested, way to (1) increase the efficiency of the research process and (2) realign R&D to address the most pressing public health problems as opposed to the most promising market opportunities. The main disadvantage of OS is the uncertainty as to how it can be financed, due to the lack of a precedent. We foresee that approaches can be refined as more projects are developed following these principles and experience accumulates.

The origin of this Essay lies in several meetings that took place to try to break this deadlock, beginning with a series of roundtable discussions [11]. There is a great deal yet to consider, debate, and try; and, as usual, the devil is in the details. This Essay is accompanied by a document (S1 Text) that lays out how the traditional structure of drug discovery and development would be impacted by an OS approach, and readers interested in the detail of the argument are directed there.

## How OS pharma might be applied

Medical R&D should respond to specific priority health needs. Opening up the R&D process to a wide range of contributors and stakeholders will allow the design of medicines that are better adapted to the needs of the end users, and will define the preferred product characteristics (PPCs) and target product profile (TPP) that will guide all the phases of product development. In OS, there are no insiders, meaning that strategic decisions may be made through informed community debate against the agreed-upon TPP. The broader community is thus involved in designing how a product will be developed, defining the studies that will be done, and establishing the criteria that will be applied for making stop/go decisions. In order to achieve this, investments are needed to build online communities and platforms that help groups collaborate effectively.

In the early stages of drug discovery, involving compound screening and analog synthesis, there is a clear argument for an open community approach. Indeed, there is early precedent in this area, such as the crowdsourcing of bioinformatics [12], compound sharing [13,14] or screening [15–17] services, open-access target validation [18], and OS medicinal chemistry campaigns [19]. Project objectives can be tackled by any interested researcher or institution, and certain research problems would be amenable to solution by crowdsourcing from large student cohorts, for example. Incentives to participate will rely on a mixture of selfless (improving a public good) and selfish (publication authorship and community acclaim) motives that are compatible with both academia and industry, where such activities could be formally sanctioned (e.g., crowdsourced undergraduate lab classes, and strategic or pro bono industry contributions) or carried out informally (e.g., strategic advice, honest brokership, and student mentorship). Such motivations have been found to govern participation in OS software projects [20]. There is a great and acknowledged potential for greater transparency in these earlier stages, which, while often termed “pre-competitive,” are nonetheless traditionally secretive.

As a candidate compound moves through development, the stakes are raised, and data become more complex and expensive to generate. The OS approach offers a number of advantages: broad stakeholder involvement has the potential to design trials that are better fit for purpose, as they include the views and requirements of both patients and health providers as to the key outcome measures, and enjoy contributions to study design and analysis by a range of experts in the field [21]. Clearly, there is an important responsibility borne by the community leaders of any OS endeavor to act as ultimate decision makers (“benign dictators” in software parlance) when a broad range of opinions are expressed or inputs received. As data are generated, there is a public-health imperative to make these data available in a timely fashion [22,23]. The continual process of transparent, unrestricted peer review of data at the heart of OS produces outcomes that may ultimately be more robust. Examples of different models whereby clinical data are being shared have started to accumulate, e.g., in the public and not-for-profit sector (the TB-Platform for Aggregation of Clinical TB Studies [TB-PACTS] for tuberculosis [24], the WorldWide Antimalarial Resistance Network [WWARN] for malaria [25], and the Infectious Diseases Data Observatory [IDDO] for Ebola [26]) as well as in the pharmaceutical industry (Clinical Study DataRequest [27]); clearly, in any such platform it is essential that patient confidentiality be protected. Transparency will help to ensure that the clinical research meets all current ethical criteria and that the appropriate standards are not compromised by any push to reduce costs.

An area in which OS could be particularly productive is drug repurposing, whereby existing drugs or candidates are found to have potential for the treatment of another disease and for which there exists a significant amount of preclinical and possibly clinical data; this is a strategy of interest for the recent Zika outbreak [28]. Should this information be shared, R&D risks, time, and costs for the new indication would be significantly reduced. A relevant example is the sleeping sickness drug candidate fexinidazole; all preclinical data were published online [29], which prompted studies in another indication (leishmaniasis) by another group [30]. Such unrestricted “reuse” of existing data is a core aspect of OS approaches and allows the rapid building of new projects from existing ones by distinct teams of researchers.

OS ultimately fosters an R&D system that is less wasteful: in the event a project is halted, all data (including negative findings) remain in the public domain, allowing similarly informed decisions by other parties in the future. It is estimated that 85% of the US$200 billion/year spent globally on health and medical research is “wasted,” primarily because information is not shared (~50% of registered clinical trials are never published in full, and ~50% of those published are not sufficiently complete for others to interpret, use, or replicate the research correctly), meaning that new studies cannot take advantage of previous research [31,32] and patients are exposed to undue risk [33].

The approval process for a drug originating from an OS process will require that drug to have an “applicant” who bears responsibility for the process in the same way as any other drug approval. While this lacks an OS precedent to our knowledge, existing PPPs or nonprofit groups could in theory shoulder this burden should a sufficiently attractive therapeutic candidate emerge, as has been the case for rectal artesunate [34]. The manufacture and distribution becomes an issue of market dynamics centered within the generics industry, facilitated by a derivative of the Creative Commons Attribution License (CC-BY), suitably adapted to pharmaceutical products, which would allow data to be shared, adapted, and used (including for the purpose of making a profit, if such a route can be found) on the sole condition that the creator is appropriately credited [35]. Openness permits reuse and is a barrier to exclusivity.

If a promising drug candidate addressing a major public health burden is generated through this process, it would be expected that significant public and philanthropic funds would be available for further development. Such is already the case in the field of poverty-related infectious diseases and neglected tropical diseases, in which there is a traditional type of what is often termed “market failure,” and other financing mechanisms, such as crowdfunding [36] and prizes [37], are being piloted. There are recent precedents of drugs being taken through such stages with government or philanthropic money and in the absence of patent protection [38], as well as an increasing number of major new funding mechanisms designed to assist with such efforts [39]. We see OS as a key means to achieve the aims of the London Declaration [40] and OS projects as good candidates for support from the proposed Pooled Fund [41]. Several innovative financing mechanisms have been created for global health [42], and these could possibly be leveraged to support global health R&D.

Yet the potential is broader. The enormous toll on our society from cancer led former United States Vice President Biden to urge researchers to “break down silos and bring all the cancer fighters together—to work together, share information, and end cancer as we know it” as part of the Cancer Moonshot initiative [43]. There are persistent calls for new approaches to the development of antibacterials to counteract the looming threat of antimicrobial resistance (AMR) [44]. Given the magnitude of the problem of dementia, it is conceivable that governments of countries in which the population is shifting progressively towards old-age citizens would be willing to invest in the development of affordable treatments (in ways that the private sector cannot) [45], rather than being overwhelmed by the eventual costs of patient care (US$226 billion in 2015 for dementia in the US alone) [46]. Consortia and funds are being established to tackle these threats without correspondingly new approaches in how the underlying research is being conducted. We see OS as providing that genuinely new approach.

## A competing model

Drug development following a traditional secretive model but funded by governments has been attempted. OS drug development (funded from whatever source) has not. Given society’s continued unmet medical needs and the scientific, efficiency, and ethical imperatives that exist to change the way in which we encourage medical innovation, there is a strong need to try alternative systems. We have the technologies to allow massive data sharing and crowdsourcing of research in real time. Furthermore, the borderless nature of OS work makes an R&D program amenable to a wide range of different funders working cooperatively, safe in the knowledge that the funding is not supporting any unnecessary duplication of effort.

One can envisage an organizational structure for OS pharma involving (1) an overarching organization that manages the legal and regulatory aspects of running projects, (2) projects themselves that are funded (by those stakeholders with the relevant resources) to achieve specific milestones, and (3) a collaboration between a funded scientific core and the wider scientific community that is able to respond to project needs by virtue of the openness of the process. Traditional funding from public or philanthropic sources could (1) leverage other investments from the private sector for specific project hurdles, and (2) stimulate significant in-kind contributions from individuals or organizations interested in the solution of a problem or demonstration of expertise. Communities of experts tend to coalesce around an OS project [47], lending it a significant research momentum that makes it competitive with more traditional closed approaches.

In November 2015, the United Nations Secretary General established a High-Level Panel to find solutions to promote innovation and access to medicines, vaccines, and diagnostics to ensure the health and well-being of all, realizing that the current commercially driven system based on intellectual property rights fails to do so [48]. The report recommended that “the world must take bold new approaches to both health technology innovation and ensuring access so that all people can benefit from the medical advances that have dramatically improved the lives of millions around the world in the last century.” If current calls for radical new approaches to solving major problems in public health are serious, then solutions that seem risky precisely because they subvert our traditional approaches should be embraced. OS pharma has that potential and should be trialed robustly as an alternative, competing model and one that brings genuinely fresh and powerful new methods to bear on our most serious public health challenges.

## Supporting information

S1 Text Article containing details of the OS pharma roadmap. This longer article originated from the first Open Source Pharma Meeting in Bellagio, Italy, July 2014.
(DOCX)

## Abbreviations

AMR: antimicrobial resistance
CC-BY: Creative Commons Attribution License
DNDi: Drugs for Neglected Diseases Initiative
IDDO: Infectious Diseases Data Observatory
MMV: Medicines for Malaria Venture
OI: open innovation
OS: open source
PDP: product development partnership
PPC: preferred product characteristic
PPP: public–private partnership
R&D: research and development
TB Alliance: Global Alliance for Tuberculosis Drug Development
TB-PACTS: TB-Platform for Aggregation of Clinical TB Studies
TPP: target product profile
WWARN: WorldWide Antimalarial Resistance Network

## References

[1] Scannell JW, Blanckley A, Boldon H, Warrington B (2012) Diagnosing the decline in pharmaceutical R&D efficiency. Nat Rev Drug Discov 11: 191–200. 10.1038/nrd3681 22378269

[2] Moon S, Bermudez J, 't Hoen E (2012) Innovation and access to medicines for neglected populations: could a treaty address a broken pharmaceutical R&D system? PLoS Med 9(5): e1001218 10.1371/journal.pmed.1001218 22615544 PMC3352855

[3] Dentico N, Ford N (2005) The courage to change the rules: a proposal for an essential health R&D treaty. PLoS Med 2(2): e14 10.1371/journal.pmed.0020014 15736991 PMC549583

[4] Incentivising research and development for the diseases of poverty (2005) Working paper of the WHO Commission on Intellectual Property Rights, Innovation and Public Health (CIPIH). http://www.who.int/intellectualproperty/submissions/IncentivisingRD.pdf. Accessed Feb 16th 2017.

[5] Goldman M, Compton C, Mittleman BB (2013) Public-private partnerships as driving forces in the quest for innovative medicines. Clin Transl Med 2:2 10.1186/2001-1326-2-2 23369569 PMC3564715

[6] Reddy D, Spigelman M (2014) Product Development Partnerships: an innovative approach to tacking neglected diseases. Development Policy Centre http://devpolicy.org/product-development-partnerships-an-innovative-approach-to-tackling-neglected-diseases-20140528/. Accessed Feb 16^th^ 2017.

[7] Lives on the Edge (2016) Médecins Sans Frontières (MSF) Access Campaign. https://www.msfaccess.org/sites/default/files/R&D_report_LivesOnTheEdge_Updated29Sept_ENG_2016.pdf. Accessed Feb 16th 2017.

[8] Maurer SM, Rai A, Sali A (2004) Finding Cures for Tropical Diseases: Is Open Source an Answer? PLoS Med 1(3): e56 10.1371/journal.pmed.0010056 15630466 PMC539047

[9] Nilsson N, Felding J (2015) Open innovation platforms to boost pharmaceutical collaborations: evaluating external compounds for desired biological activity. Future Med Chem 7:1853–1859. 10.4155/fmc.15.122 26393392

[10] Årdal C, Alstadsæter A, Røttingen JA (2011) Common characteristics of open source software development and applicability for drug discovery: a systematic review. Health Res Policy Syst 9:36 10.1186/1478-4505-9-36 21955914 PMC3206459

[11] Open Source Pharma. http://www.opensourcepharma.net/. Accessed March 31st 2016

[12] Bhardwaj A, Scaria V, Raghava GPS, Lynn AM, Chandra N, Banerjee S, et al (2011) Open source drug discovery—a new paradigm of collaborative research in tuberculosis drug development. Tuberculosis 91: 479–486. 10.1016/j.tube.2011.06.004 21782516

[13] Van Voorhis WC, Adams JH, Adelfio R, Ahyong V, Akabas MH, Alano P, et al (2016) Open source drug discovery with the Malaria Box Compound Collection for neglected diseases and beyond. PLoS Pathog 12(7): e1005763 10.1371/journal.ppat.1005763 27467575 PMC4965013

[14] Arrowsmith CH, Audia JE, Austin C, Baell J, Bennett J, Blagg J, et al (2015) The promise and peril of chemical probes. Nat Chem Biol 11: 536–541. 10.1038/nchembio.1867 26196764 PMC4706458

[15] The National Cancer Institute Development Therapeutics Program (DTP). https://dtp.cancer.gov/. Accessed Feb 16th 2017.

[16] The Community for Open Antimicrobial Drug Discovery, http://www.co-add.org/. Accessed Feb 16th 2017.

[17] EU-OPENSCREEN. http://www.eu-openscreen.eu/. Accessed Feb 16th 2017

[18] Lee WH (2015) Open access target validation is a more efficient way to accelerate drug discovery. PLoS Biol 13(6): e1002164 10.1371/journal.pbio.1002164 26042736 PMC4456377

[19] Williamson AE, Ylioja PM, Robertson MN, Antonova-Koch Y, Avery V, Baell JB, et al (2016) Open Source Drug Discovery: Highly Potent Antimalarial Compounds Derived from the Tres Cantos Arylpyrroles. ACS Cent Sci 2: 687–701. 10.1021/acscentsci.6b00086 27800551 PMC5084075

[20] Roberts DA, Hann IH, Slaughter SA (2006) Understanding the motivations, participation and performance of open source software developers: a longitudinal study of the Apache projects. Manage Sci 52: 984–999.

[21] Sablinski T (2014) Opening up clinical study design to the long tail. Sci Transl Med 6: 256ed19 10.1126/scitranslmed.3009116 25273093

[22] The AllTrials Campaign. http://www.alltrials.net/. Accessed Feb 16th 2017

[23] Moorthy VS, Roth C, Olliaro P, Dye C, Kieny MP (2016) Best practices for sharing information through data platforms: establishing the principles. Bull World Health Organ 94:234–234A. 10.2471/BLT.16.172882 27034512 PMC4794314

[24] TB-platform for Aggregation of Clinical TB Studies (TB-PACTS), Critical Path Initiative. http://www.cptrinitiative.org/tag/tb-pacts/. Accessed Feb 16th 2017.

[25] Worldwide Antimalarial Resistance Network. http://www.wwarn.org/about-us. Accessed Feb 16th 2017.

[26] Infectious Diseases Data Observatory. https://www.iddo.org/data-sharing. Accessed Feb 16th 2017.

[27] Clinical Study DataRequest.com. https://clinicalstudydatarequest.com/. Accessed Feb 16th 2017.

[28] Kincaid E (2016) A second look: Efforts to repurpose old drugs against Zika cast a wide net. Nat Med 22(8): 824–825. 10.1038/nm0816-824 27490430

[29] Torreele E, Bourdin Trunz B, Tweats D, Kaiser M, Brun R, Mazué G, et al (2010) Fexinidazole—a new oral nitroimidazole drug candidate entering clinical development for the treatment of sleeping sickness. PLoS Negl Trop Dis 4(12):e923 10.1371/journal.pntd.0000923 21200426 PMC3006138

[30] Wyllie S, Patterson S, Stojanovski L, Simeons FR, Norval S, Kime R, et al (2012) The anti-trypanosome drug fexinidazole shows potential for treating visceral leishmaniasis. Sci Transl Med 4:119re1 10.1126/scitranslmed.3003326 22301556 PMC3457684

[31] Chalmers I, Glasziou P (2009) Avoidable waste in the production and reporting of research evidence. Lancet 374: 4–10. 19525005 10.1016/S0140-6736(09)60329-9

[32] Chalmers I, Glasziou (2016) Is 85% of health research really “wasted”? Newsletter of the International Society for Evidence-based Heath Care, 22:3–5, available at http://www.isehc.net/wp-content/uploads/2011/12/22nd-Edition1.pdf. Accessed Feb 16th 2017.

[33] Gøtzsche PC (2011) Why we need easy access to all data from all clinical trials and how to accomplish it. Trials 12:249 10.1186/1745-6215-12-249 22112900 PMC3264537

[34] Malaria treatment rectal artesunate to be developed in India, World Health Organization 2014 http://www.who.int/tdr/news/2014/malaria-treatment/en/. Accessed Feb 16^th^ 2017.

[35] Carroll MW (2013) Creative commons and the openness of open access. N Engl J Med 368: 789–791. 10.1056/NEJMp1300040 23445090

[36] Dragojlovic N, Lynd LD (2014) Crowdfunding drug development: the state of play in oncology and rare diseases. Drug Discov Today 19:1775–1780. 10.1016/j.drudis.2014.06.019 24973645

[37] The Longitude Prize. https://longitudeprize.org/. Accessed Feb 16th 2017.

[38] Bompart F, Kiechel JR, Sebbag R, Pécoul B (2011) Innovative public–private partnerships to maximize the delivery of anti-malarial medicines: lessons learned from the ASAQ Winthrop experience. Malar J 10: 143 10.1186/1475-2875-10-143 21605364 PMC3117751

[39] National Center for Advancing Translational Sciences (NCATS). https://ncats.nih.gov/. Accessed Feb 16th 2017.

[40] London Declaration, Uniting to Combat Neglected Tropical Diseases (2012). http://unitingtocombatntds.org/london-declaration. Accessed Feb 16th 2017

[41] Special Programme for Research and Training in Tropical Diseases (TDR) (2016). Health Product Research and Development Fund: a Proposal for Financing and Operation. http://www.who.int/tdr/publications/r_d_report/en/. Accessed Feb 16th 2017

[42] Atun R, Knaul FM, Akachi Y, Frenk J (2012) Innovative financing for health: what is truly innovative? Lancet 380: 8–14. 10.1016/S0140-6736(12)61460-3 23102585

[43] Biden J (2016) Inspiring a New Generation to Defy the Bounds of Innovation: A Moonshot to Cure Cancer, Medium. https://medium.com/@VPOTUS/inspiring-a-new-generation-to-defy-the-bounds-of-innovation-a-moonshot-to-cure-cancer-fbdf71d01c2e#.co6nn3z7q. Accessed Feb 16th 2017

[44] Payne DJ, Miller LF, Findlay D, Anderson J, Marks L (2015) Time for a change: addressing R&D and commercialization challenges for antibacterials. Philos Trans R Soc Lond B Biol Sci 370: 1670. 10.1098/rstb.2014.0086 PMC4424435 25918443

[45] Lo AW, Ho C, Cummings J, Kosik KS (2014) Parallel discovery of Alzheimer’s therapeutics. Sci Transl Med 6: 241cm5 10.1126/scitranslmed.3008228 24944190

[46] Alzheimer's Association (2015) 2015 Alzheimer’s Disease Facts and Figures. Alzheimers Dement 11: 332–384. 25984581 10.1016/j.jalz.2015.02.003

[47] von Krogh G, Spaeth S, Lakhani KR (2003) Community, joining and specialization in open source software innovation: a case study. Res Policy 32: 1217–1241.

[48] The United Nations Secretary-General’s High-Level Panel on Access to Medicines, Final Report. http://www.unsgaccessmeds.org/final-report/. Accessed Feb 16th 2017

