# Supplementary material for: An open source pharma roadmap
Source: PLoS Med. 2017 Apr 18;14(4):e1002276. doi: 10.1371/journal.pmed.1002276 (PMC5395155; doi:10.1371/journal.pmed.1002276)
Supplement: S1 Text — This longer article originated from the first Open Source Pharma Meeting in Bellagio, Italy, July 2014. (DOCX) [file pmed.1002276.s001.docx]

**Supporting Information**

**for**

***An Open Source Pharma Roadmap***

Manica Balasegaram^1^, Peter Kolb^2^, John McKew^3a^, Jaykumar Menon^4^, Piero Olliaro^5,6^, Tomasz Sablinski^7^, Zakir Thomas^8b^, Matthew H. Todd^9^*, Els Torreele^10^, John Wilbanks^11^

1 Médecins Sans Frontières, Geneva, Switzerland

2 Department of Pharmaceutical Chemistry, Philipps-University Marburg, Marburg, Germany

3 National Institutes of Health, National Center for Advancing Translational Sciences (NCATS), Bethesda, Maryland, United States of America

4 McGill University Institute for the Study of International Development, Montreal, Canada

5 UNICEF/UNDP/World Bank/WHO Special Programme for Research and Training in Tropical Diseases (TDR), World Health Organization, Geneva, Switzerland

6 Centre for Tropical Medicine and Global Health, Nuffield Department of Clinical Medicine, University of Oxford, Oxford, United Kingdom

7 Transparency Life Sciences, New York, New York, United States of America

8 Open Source Drug Discovery, Council of Scientific and Industrial Research (CSIR), New Delhi, India

9 School of Chemistry, The University of Sydney, Sydney, Australia

10 Public Health Program, Open Society Foundations, New York, New York, United States of America

11 Sage Bionetworks, Seattle, Washington, United States of America

a Current: Lumo Pharma, Austin, Texas, United States of America

b Current: Directorate of Risk Assessment, Government of India, New Delhi, India

* matthew.todd@sydney.edu.au

**Abstract**

Alternative approaches for discovering and developing new medicines have been proposed that attempt to solve persistent weaknesses of the current pharmaceutical system. Many such alternatives involve greater degrees of openness and sharing. We believe a model that has been suggested and piloted but never fully evaluated is open source pharma (OSP), in which all data and ideas are shared, anyone may participate and there are neither patents nor monopolies. In this paper we describe how OSP could be made to work. The research mechanism is less akin to the classical “chevron” structure of drug discovery (in which great risk is assumed by early participants) and more like a mosaic model in which diverse participants may play a role that is made clear through the model’s complete transparency. Non-traditional financing, much from public funds, would be required, and efficient use of such funds would be ensured through the continual peer review enabled by the openness of all project components.

Part I – The **Why**

Key points:

a) There is a clear and pressing need for models of drug discovery and development that both complement and address the inadequacies of the current model.

b) A multitude of alternative approaches has been proposed and implemented, many of which rely on a closed research process.

c) The current model is inefficient because it does not prioritize the real public health needs, often excludes appropriate partners who can contribute to the process, and often duplicates efforts – all of which raise costs.

**Background**

1.0 The pharmaceutical industry has a history of producing therapeutics but in its current incarnation is failing to meet society’s needs on its own.^[[1]](#endnote-1)^ With the current contraction of the industry comes a declining number of distinct scientific approaches to solving problems associated with human health.^[[2]](#endnote-2),^^[[3]](#endnote-3)^

1.1 The pressures arising from the way the pharma industry is financed have led to suggestions that pharma’s current business model is centered on the benefit to shareholders rather than patients.^[[4]](#endnote-4),^^[[5]](#endnote-5),^^[[6]](#endnote-6),^^[[7]](#endnote-7),^^[[8]](#endnote-8)^

1.2 In an ideal world one would wish to see a combination of sustainable, perhaps even profitable, financing of pharmaceutical projects aimed at health outputs that meet the most pressing needs of the most people, generating affordable medicines.

**Existing Alternatives**

2.0 In recent years, several alternatives to the operations and financing of the current pharma model of discovering and developing new medicines have been proposed,^[[9]](#endnote-9)^ including:^[[10]](#footnote-1)^

2.1 Product Development Partnerships (PDPs), which have had a significant impact on marshaling global efforts (across traditional academia and industry) in the more neglected diseases (*e.g.,* DNDi, MMV, TB Alliance).^[[11]](#endnote-10)^ Related to these are non-profit groups focused on specific diseases that push a research agenda sometimes *via* experimental science and advocacy (*e.g.,* the ALS Therapy Development Institute).

2.2 Closed Consortia, which have arisen in an attempt to increase the efficiency of the relevant biomedical research (*e.g.,* the Accelerating Medicines Partnership, The TB Drug Accelerator, The Structure-guided Drug Discovery Coalition, TransCelerate).

2.3 Virtual Pharma Companies that adopt various models driving collaboration between a central organization (holding IP) and contract research organizations/academia, typically focused on the early stages of drug discovery.^[[12]](#endnote-11)^

2.4 “Open Innovation” platforms that aim to increase collaborative work between sectors while maintaining control of IP through novel licensing arrangements (*e.g.,* Eli Lilly’s Open Innovation Drug Discovery, Pfizer’s Centers for Therapeutic Innovation, GSK’s Discovery Fast Track Challenges, Bayer’s Drugs4Targets). It could be said that the expansion of pharma activities into precompetitive space (target validation, hit-finding collaborations with academia) arises from the failure of the current system to generate new therapeutics at the rate required of the industry’s business model.

2.5 Alternative financing concepts,^[[13]](#endnote-12)^ in which the cost of R&D does not need to be financed by the sales of medicines (colloquially called “de-linkage”)^[[14]](#endnote-13),^^[[15]](#endnote-14)^, for example or the use of prizes or levies on airline tickets.^[[16]](#endnote-15)^

2.6 Advance Market Commitments (AMCs), aimed at de-risking investment through promise of a purchase should a product be developed and produced; this has been used by GAVI, the vaccine alliance, in conjunction with long-term government bonds arising from an International Finance Facility that provides a long-term financing stream.^[[17]](#endnote-16),^^[[18]](#endnote-17),^^[[19]](#endnote-18),^^[[20]](#endnote-19)^

2.7 Priority Review Vouchers (PRVs), in which accelerated review may be granted for a product of choice to (and sold by) a company that takes a therapeutic for a neglected disease to market;^[[21]](#endnote-20),^^[[22]](#endnote-21)^ the scheme’s critics point to instances where existing therapeutics developed with public money have successfully been used to generate PRVs.^[[23]](#endnote-22)^

2.8 There is a great variety of other financial initiatives under development,^[[24]](#endnote-23),^^[[25]](#endnote-24)^ but we limit ourselves in this section to considering those that have to date been implemented.

2.9 Patent pooling, aimed at permitting the straightforward licensing of patented material for specific uses.^[[26]](#endnote-25),^^[[27]](#endnote-26),^^[[28]](#endnote-27)^

2.10 Last but not least, biomedical R&D (in particular for neglected health needs) has been heavily supported by public and philanthropic sources, including direct grants, trial platforms, capacity strengthening, tax breaks, etc. These include government agencies and funding programmes (*e.g.,* US NIH, EU research programmes), special programmes (*e.g.,* TDR, EDCTP), and private foundations (e.g. BMGF, Wellcome Trust).

2.11 It is likely that there will be no single solution to the problem at hand, but that a combination of push (*e.g.,* grants), pull (*e.g.,* prizes) and pooling methods will need to be used^[[29]](#endnote-28)^ depending on the disease addressed.

**Chevron Structure and Secrecy**

3.0 We suggest a more radical alteration in the underlying way we discover and develop medicines.

3.1 Many current methods or suggestions for fixing the problem derive from alterations to the classic “chevron” diagram of drug discovery and development - a 10+ year linear construction of discovery, development, clinical and distribution phases that has typically been managed by a single company or a small collection of entities. The monolithic process has been the responsibility of one agent, which assumes all the risks and expects high financial returns as reward (usually through intellectual property control to guard the required financial exclusivity at the end).

3.2 The inefficiency of this approach is its secrecy.

3.2a Others, well qualified to work on the problem, are excluded because the composition of the team is both restricted and largely defined in advance.

3.2b Unproductive lines of enquiry are not shared (or are found to be too difficult to publish), leading to expensive duplications of effort and a de-emphasis of the importance of negative data, or a level of detail that ensures reproducibility.^[[30]](#endnote-29),^^[[31]](#endnote-30)^

3.2c Research programs in academia, but particularly industry, which have been deprioritized for financial or strategic reasons may suffer significant losses to the archived data (particularly if a team is disbanded) making it a challenge to resurrect such programs in the future.

3.2d The patient community is not typically engaged in the process of development. An emerging outlier here is rare disease research, for example the funding of Kalydeco by the Cystic Fibrosis Foundation, and the Leukemia and Lymphoma Society Therapy Acceleration Programs.

3.2e Geniuses stand on the shoulders of giants. If we acknowledge that science is a collective enterprise driven by critical engagement with others, then walls placed around ideas should not be seen merely as economically necessary inconveniences but as obstacles that compromise the ability of science to deliver to society most effectively.

**Modifications to the Structure of Pharma**

4.0 A solution would be to abandon the chevron structure and rebuild it as a networked model (a “mosaic”) where small components of the whole are addressed by those best placed to do so using funding and expertise from diverse sources as needs arise.

4.1 A relatively small project kernel could serve as a means to coordinate and make decisions based on inputs from the broader community.

4.2 The contributions would ideally be able to self-assemble around opportunities and roadblocks because all participants would be up to date on project requirements.

Part II – The **What**

Key points:

a) An alternative approach, Open Source Pharma, involves the complete sharing of all data and ideas, the possibility for anyone to participate in the research, and the exclusion of monopolies.

b) The level of transparency of this approach distinguishes it from other collaborative approaches variously described as “open innovation”.

c) Though the concept of OSP was proposed a decade ago, and there have been pilot projects examining feasibility, OSP has never been properly evaluated.

**An Open Source Approach**

5.0 Such modifications are possible by adopting an inclusive approach that we will call “open source” pharma (OSP).

5.1 In essence the term “open source”, derived from the software industry, means that the data and ideas in a project are freely shared (and may be reused) and anyone may participate at any level. Contributors are kept up to date *via* a process of radical transparency.

5.1a There is an obvious distinction between software (involving code) and pharma (involving physical objects). There remains the essential similarity - that “open source” is a description of an inclusive way of working where the “code” in therapeutic development - the how and why of chemical synthesis, or the methods and results of a clinical trial - may be seen in detail, reused and responded to in real time, making the “playing field” of participation level.

5.2 The structure of the mosaic means that a component may be completed and the project paused if no resources or partners are available to pursue it, but then resumption is relatively straightforward given that all project data are freely available in the public domain. Open source research is therefore more time-resistant than other research strategies. Roadblocks would attract specialists who know their work will impact the project since all work is openly disclosed and unnecessary duplication is not a risk; similarly the smaller size of the necessary components of the mosaic reduces the risk of commitment by each participant.

**Previous/Current Examples of Open Methods in Drug Discovery and Development**

6.0 Open source drug discovery was mooted more than 10 years ago.^[[32]](#endnote-31),^^[[33]](#endnote-32),^^[[34]](#endnote-33)^

6.1 Towards open source drug discovery, there have been significant efforts over the years in biomedical data sharing^[[35]](#endnote-34),^^[[36]](#endnote-35),^^[[37]](#endnote-36)^, and more recently in the sharing of physical samples to aid collaborative research.^[[38]](#endnote-37),^^[[39]](#endnote-38),^^[[40]](#endnote-39)^

6.2 A small number of recent projects have exemplified what is involved in crowd-sourced or open source drug discovery mechanisms, i.e. those addressing new models for discovery and development phases.^[[41]](#endnote-40),^^[[42]](#endnote-41)^ Such projects have demonstrated the effectiveness of various software components (bespoke or off-the-shelf) able to host distributed, data-rich collaborations in the open domain and recruit expert participation.^[[43]](#endnote-42),^^[[44]](#endnote-43)^ for example in the first crowd-sourced annotation of the TB genome, performed by the Open Source Drug Discovery (OSDD) group in India.^[[45]](#endnote-44)^ The Open Source Malaria consortium for example has operated since 2011 on an explicit set of 6 “Laws” that align with the above-mentioned mechanism of Open Source Pharma (Figure 1).


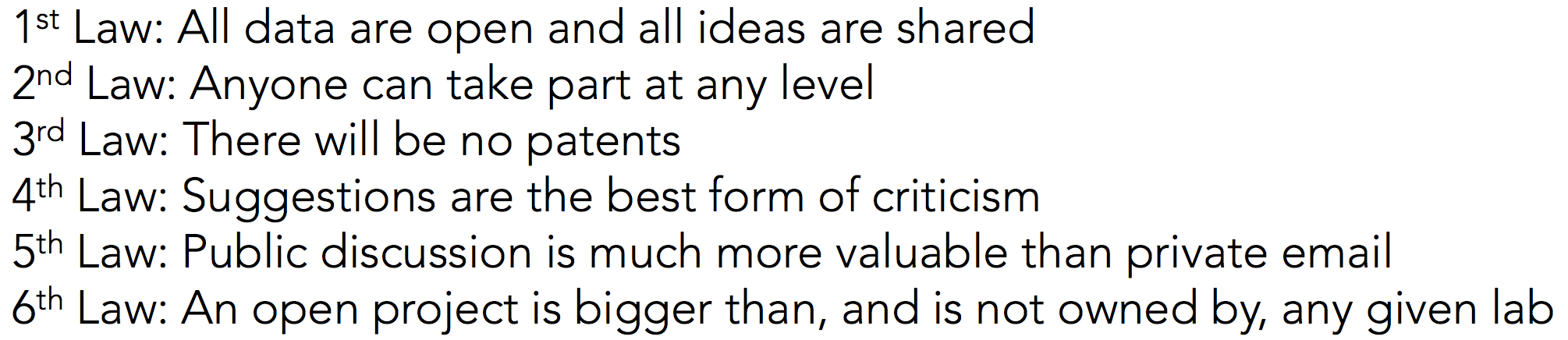


**Figure 1**. Operating Principles of the Open Source Malaria Consortium^[[46]](#endnote-45)^

6.3 There are frameworks in place for collaborative data sharing in bioinformatics and for the discovery of chemical probes relevant to chronic disease where the intention is to take compounds through to a proof of clinical mechanism in the absence of intellectual property protection.^[[47]](#endnote-46),^^[[48]](#endnote-47)^

7.0 Building on these important precedents, this paper examines how the classic chevron diagram may be reconstructed as a nimble network of diverse participants, including virtual pharma companies, government laboratories, open source groups, contract research organisations (CROs), patients and others.

7.1. There exist some projects already which combine several of these features, providing further precedent for the interoperability of the components and some testament to the outdated conception of the traditional chevron structure.

7.2 Throughout the open source structure traditional IP is not sought since this is part of a closed model where the flow of ideas and participation by the community is limited in anticipation of patent protection. Generally, this is contrary to the policies of most pharma companies and university technology transfer offices.

Part III – The How

Key points:

a) Adapted target product profiles (TPP) for priority medicines will be generated through crowd-sourcing.

b) Open source projects will have clearly-identified requirements reflecting the TPP, permitting scientific and financial contributions from a wide range of people and organizations.

c) Crowd-sourcing and crowd-funding may be employed to solve technical and financial roadblocks, such as molecule synthesis or the optimization of clinical trial design.

d) Financing of OSP will need to come from public, philanthropic and private sources. Alternative business models regarding R&D will be required to stimulate private sources of funding. As a general principle, public and philanthropic funding should demand data sharing and transparency.

**Feasibility of Open Source Principles Along the Traditional Chevron Pipeline**

**8 Discovery and Development Phases**

8.0 The discovery of hit compounds and their transit through lead optimization is perhaps the cleanest possible application of a process that would benefit from inputs of the crowd, rendering it amenable to an open source approach.^[[49]](#footnote-2)^

8.1 The construction of Target Product Profiles (TPPs) will benefit from the input in the open domain of a broad range of stakeholders.^[[50]](#endnote-48)^

8.2 Recent phenotypic screens have discovered thousands of experimental hits requiring resynthesis and exploration, and it has been possible to transfer these data to the public domain efficiently using open databases. That many of these screens have been carried out by industrial organizations is testament to the importance of such datasets to stimulate research activity by a community and the relatively low expense of conducting such screens *vs*. the extent of downstream activity they catalyze.^[[51]](#endnote-49)^

8.3 More rational modeling approaches (genomics through to computer-aided drug design) can benefit from inputs from a network of computer scientists.

8.4 Such projects can be conceptually modularized, for example through the community construction of a shortlist of 50 compounds requiring synthesis, or a set of small molecule-protein interactions under consideration in a repurposing project. Given the costs associated with human-led synthetic chemistry, and the number of compounds typically required in a hit-to-lead campaign through several iterations of synthesis and evaluation, it is important that the larger open network is used efficiently to accelerate synthesis of the right molecules. The component tasks can be apportioned to multiple participants working as:

8.4a paid research students, *i.e.* as part of PI-led grants or university degree programs relevant to the larger project, perhaps trialing or showcasing new scientific methods. Responsiveness to high-value or stubborn project needs could be achieved through micro-grants to those undertaking specific tasks if no funds exist already in related projects.

8.4b science undergraduates (of which there are hundreds of thousands globally) conducting laboratory classes for which they receive academic credit or some other microattribution. Such mechanisms have already been used for synthetic chemistry in open^[[52]](#endnote-50)^,^[[53]](#endnote-51)^ and closed^[[54]](#endnote-52)^ projects. Sophisticated microattribution mechanisms are well-established in software communities, providing a quantitative proxy for reputation in academic/scientific circles.

8.4c volunteers interested in gaining research experience or building their CVs.

8.4d larger-scale inputs of organizations inherently able to participate (*e.g.,* National Center for Advancing Translational Sciences (NCATS), the European Lead Factory) or those such as pharmaceutical companies interested in showcasing corporate social responsibility (of employees or the organization as a whole), or to showcase in detail technical expertise to potential clients and collaborators.

8.5 Efficient use of this network of participants is possible because the network is open, meaning there is no unnecessary duplication and the participants may learn from each other by, for example, immediately disseminating effective protocols.

8.6 The software required to permit elementary communication between the participants exists,^45,46,^^[[55]](#endnote-53)^ though significant improvements are needed in this sector to ensure optimal use of the crowd’s resources as well as permanence and linkage of the disparate outputs.

8.7 Brute force effort from an army of chemists would be counterproductive without planning. Careful management and encouragement of students is required to ensure quality control, a focus on profitable scientific lines of enquiry and no “orphaning” of side projects in what would become a large amount of data. Such mentorship can arise from the immediate supervisors of students, through peer-to-peer instruction, from assigned mentors in PDPs and from the largely untapped community of retired scientists keen to maintain contact with ongoing research projects.

8.8 Such mentorship will require some formalization, and could present extensive opportunities for a new “*pro bono*” approach to pharma that represents such a powerful force in the legal sector.^[[56]](#endnote-54)^

8.9 Ideas for molecules or avenues of enquiry that are seen to be unproductive by a majority of participants and then mothballed would nevertheless live on in open projects since they remain in the public domain, rather than orphaned in reports or on local storage, and may be pursued later by those wishing to do so. It is important for the data generated in this phase to be machine-readable (through the use of trivial chem- or bioinformatic strings, for example) so that such “parked” ideas can be found and cross-correlated rather than re-invented.

8.10 Assays for biological activity, using openly-agreed protocols, would need to include adequate controls to ensure inter-laboratory reproducibility, though in some cases use of a single testing laboratory may present benefits that offset the expense of compound shipment. Wider, detailed consultation on persistently challenging protocols (*e.g.,* the assay for potency *vs*. *Mycobacterium tuberculosis*) would likely be of general benefit to reproducibility in the respective fields.

8.11 If inputs are needed for which no contributor may be found in the short term (*e.g.,* a bioisosteric replacement in a hit-to-lead campaign requiring a challenging synthesis), various motivations may lead to such a roadblock being overcome, including:

8.11a Acclaim from the community at solving what others could not, coupled with the promise of publication authorship.

8.11b A prize that is crowdfunded or contributed by a company, learned organization, PDP or some combination of these.^[[57]](#endnote-55)^ The prize could be constructed to reward contributions for increases in efficiency of sub-projects (milestone prizes), rather than entirely for the bigger solution alone; in either case the conditions under which money is released would need to be carefully defined. Prize systems that are “closed”^[[58]](#endnote-56),^^[[59]](#endnote-57)^ would be disfavoured compared to “open” prize systems in which each submitted solution can be immediately seen and reused by all participants, a strategy that has provided efficient solutions recently in various fields^[[60]](#endnote-58),^^[[61]](#endnote-59)^ but which has been used as far back as the 18th Century.^[[62]](#endnote-60)^ A competition operating openly brings an intriguing possibility: that a post mortem is possible to determine the relative contributions of (and rewards to) the participants, as opposed to determining who first crossed the finishing line.

8.11c Direct payment to a CRO, or through an open bidding process for inputs of the molecule or close analogs^[[63]](#endnote-61)^ that could be based on existing structures of assay marketplaces (*e.g.,* Assay Depot, Science Exchange) in which project needs would be openly described. A “post what you need” service becomes powerful when combined with an open philosophy since the greatest number of potential suppliers and collaborators may see the need and bid competitively.

8.11d Engagement of existing state- or consortium funded network expertise prioritized through peer review (*e.g.,* the EU Lead Factory)

8.12 Stop-go decision should be made transparent though a crowd-based exercise. It is tempting to think this would lead to a choir of voices preventing a clean decision, whereas in reality (and in the experience of some of the authors) a natural hierarchy of experience means some voices naturally carry more weight than others. The open availability of both the decision itself and the data behind the decision limit the possibility of decision-making based on personal or economic bias. The intellectual and logistical cost of “forking” a project in the event of a disagreement lessens the risk that the community divides, though, again, possible project branches always remain available for future re-adoption in open source projects.

**Lead Optimization and Preclinical**

9.0 There are no examples of open source efforts from the point of lead optimization forward through to preclinical work, where the costs per compound series rise sharply.

9.1 *In vitro* metabolic studies, *in vivo* pharmacokinetic studies, API process chemistry optimization, GLP toxicology (including development of validated assays for rat and dog and eventually humans to enable a clinical trial) and genotoxicity studies are examples of component studies that would always be needed for any compound regardless of the research mechanism.

9.2 On the assumption that such studies would not be provided *pro*- or *lo-bono*, these would need to be paid for.

9.3 A network of CROs or specialist university centers exists for all this work already, so may be brought in on demand to complete the work quickly. Costs tend to be competitive since the techniques employed are frequently generic, particularly for *in vitro* studies. There are existing programs directed to bridging this gap through resource access, contract access, or direct grants, and these could be deployed here (*e.g.,* the NCATS Therapeutics for Rare and Neglected Diseases (TRND) and Bridging Interventional Development Gaps (BrIDGs) programs, the NIH Blueprint Neurotherapeutics Network, and the National Heart, Lung and Blood Institute Science Moving Towards Research Translation and Therapy (SMARTT) program).

9.4 The costs would need to be met, on a case-by-case basis, by the organization(s) sponsoring the series.

9.5 The data and methods should be shared with, and analyzed by, the community, as has already occurred in the OSM consortium where some costs have been met by the sponsoring agency. Thus while there is less need for experimental input from the broader community at this stage, there is advantage in protocol design and data interpretation by specialized communities as a form of distributed quality control, drawing on the experience of previous campaigns in which those people may have been involved.

9.6 The clinical trial material and preclinical formulation work could benefit significantly from a crowd-sourced approach given that there are many potential participants, ranging from academic laboratories through to CROs, and given the size of the matrix of possible experimental approaches. The process chemistry, which would be defined at this stage, is amenable to non-experimental approaches (“armchair mode”).

10.0 Plotting of the clinical strategy may also take place openly and involve the patient/advocacy communities. Biomarkers will need to be developed so they can be validated through Phase 2 and there are examples of the sharing of biomarker strategies in a consortium approach (*e.g.,* the Foundation for the National Institutes of Health Biomarkers Consortium).^[[64]](#endnote-62)^

10.1 At the pre-IND meeting with the regulatory authority the therapeutic must be represented by a sponsor willing to be responsible for the community’s compound.

10.2 The considerable personpower needed to manage the paperwork of a filing is moderately suited to a crowd-based effort, though there exist commodity services for this part of the pipeline, and *ca.* 90% of the filing consists of documents that would already be in the public domain.

**Clinical**

11.0 There are scientific and practical advantages, in addition to an ethical imperative, in involving a range of stakeholders (both researchers and end-users such as health-care providers, policy-makers, and patients) in the clinical trial process. For example, a call in 2015 from WHO around trial design for cutaneous leishmaniasis sought such advantages.^[[65]](#endnote-63)^

11.1 It is of paramount import that all trials be registered, protocols and analytical plans published, and results be made available in a timely fashion, whether positive or negative.^[[66]](#endnote-64),^^[[67]](#endnote-65)^

11.2 New information technologies such as telemonitoring and mobile phone applications have reduced the costs of delivering health care services. It is reasonable to expect that these technologies, which will be particularly amenable to an open source approach, will also reduce the cost of clinical trials.

11.3. In principle, the advantages of OSP can be applied to all phases of clinical development (Phase 1, 2 and 3) as well as post-registration studies (Phase 4, intervention trials).

11.4. This includes a transparent process of obtaining input of the different stakeholders in the clinical trial design and statistical analytical plan, and data-, process- and knowledge-sharing (*e.g.,* sharing of protocols, standard procedures, anonymized patient data).

11.5 Significant public and philanthropic funding should be available for clinical development. Again the outputs should be made freely available and the crowd could assist with the statistical analysis and report writing in line with the strict existing regulatory requirements.

11.6 The execution of a clinical trial designed by the crowd would be carried out in the same way as the current system, with an academic or other hospital-based PI. The sponsor and PI would have ultimate responsibility for the conduct of the study, recruiting patients, assuring their safety and creating high quality data.

11.7. Where significant barriers arise to funding this portion of the pipeline, push/pull mechanisms could be explored. The range of such measures include options that have already been used (see above), as well as new alternative mechanisms of rewarding R&D. Many existing mechanisms, however, have not been designed to function with an open source research process.

12.0 Phase II is the crucial stage of development for any new compound and requires the most thorough discussions on design. The choice of dose, inclusion criteria, duration of treatment *etc.* made prior to entering Phase 2 often determines the “make or brake” of a drug. Unfortunately in the current development paradigm the discussion can be inadequately deep or broad and consequently many compounds advance to Phase 3 only to fail after a few years with many millions of dollars spent and the unnecessary exposure of patients. In addition, the development of many compounds is discontinued based on “failed” Phase 2 experiments where in fact the culprit was probably poor design. For these reasons the contribution of patients and researchers to the design of clinical trials is important at the entry to, and during, Phase 2 clinical development. There are already examples of crowd-sourced design in this phase in multiple sclerosis, prostate cancer and orphan indications.^[[68]](#endnote-66),^^[[69]](#endnote-67)^

12.1 The nature and extent of the openness of the design and analysis of any Phase III trial would depend on the disease under study. Avoidance of bias is important at all stages of clinical development, and it is critical in Phase III. In some cases continuation of the discussion of a study to the execution stage may endanger randomization or facilitate unblinding, both fatal for study integrity. These dangers are real and need to be considered in the context of an indication, patient population, geographical distribution *etc.* before the decision as to the degree of openness to public discourse is made. Regardless, the instant availability of data in the public domain after study completion should not be compromised. Successful and failed trial results contribute to the generation of knowledge and ultimately benefit not only society at large but also commercial sponsors of drug development who are currently reluctant or overtly oppose the sharing of data except where they are forced to do so by regulatory requirements.

12.2 The submission of the NDA dossier is largely commodity work that may be outsourced, though a substantial portion could be carried out by the crowd in condensing the large amount of information required for the dossier into a readable summary for the regulatory agencies. When the dossier is finally submitted, it must meet all the standards and demands of the regulatory authorities; the quality of the document can influence the decision by the agency and the labeling of the product.

12.3 Flexible regulatory approaches already in place (*e.g.,* fast-track review for breakthrough therapy designations) can be used to accelerate clinical development, however it needs to be recognized that significant data are still required to guide policy and practice beyond registration, For this reason OSP is critical to the whole value chain, beyond R&D.

**Post-approval**

13.0 In order to make the medicine available, it must be manufactured at a large enough scale to cover needs. A plant would have been identified through a transparent review and selection process earlier during development, and inspected/approved by a regulatory agency.

13.1 The generics industry^[[70]](#endnote-68)^ can operate with little or no intellectual property protection, providing a model for the late process synthesis stage, again with freely shared data on output quality. Additional margin is possible through branding.

14.0 The price of the medicine should be set through a transparent process. Considering that development would have been primarily financed through public and philanthropic sources, making the product a public good, pricing should reflect cost of manufacturing plus a modest mark-up.

14.1 The logistics of getting the medicine to the patients who need it most would be a consultative process between agencies with information on the location of those most in need and an understanding of the physical and regulatory environments in each country, but this process would benefit from sharing of data and harmonization of approaches. There would be straightforward opportunities to highlight instances of governments delaying access to much-needed medicines because of regulatory inefficiency.

14.2 There would be significant advantages in crowd-sourcing new approaches to conducting Phase IV and more implementation-type studies. The open model (again including a range of stakeholders) would provide a new forum for working together on important issues in the real world at this phase. A similar approach could be used to identify innovative and more effective ways of monitoring safety of medicines that also reflect patient needs, to complement the classical model of pharmaco-vigilance.

**Financial and Legal**

15.0 The principle of open source and data sharing inherently precludes monopoly protection (and therefore removes the usefulness of the patent pool concept, for example). The inevitable question is: How will R&D be financed given there is no IP?

15.1 Open source should not be confused with “free of charge”; open source can both consume and generate significant financial resources. As a general rule, however, the financing of R&D is generally not achieved through rent-seeking from a derived product of that R&D.

15.2 Global expenditure on biomedical R&D is split roughly 2:1 between industry:public sources, yet the average public figure is substantially less than 1% of GDP.^[[71]](#endnote-69)^ However, according to the not-for-profit organization *Association Mieux Prescrire*, in the current model 75% of the new medicines that reach the market present no added therapeutic value.^[[72]](#endnote-70)^

15.3 As mentioned above, the networked structure of components means that necessary costs can be met from diverse sources as needs arise, as opposed to financial support for the whole process from a single organization. The open structure allows projects to remain active even if there is a delay in funding, since all participants in a given campaign are aware of project progress, and, with effective curation and leadership, it will be clear what is needed at each point. Advertising such needs to the public can then bring in specialist participants, unknown to the existing team, without long-term obligation to the rest of the development process, a mechanism that has already been demonstrated in an open source project in neglected tropical diseases sponsored by the World Health Organization;^[[73]](#endnote-71)^ prizes should also be effective in doing this.

15.4 The leveraging of community resources at minimal additional cost in open source projects presents granting agencies with a particularly efficient use of their resources.

15.5 There are historical examples of therapeutics brought to the market in the absence of IP protection, such as penicillin^[[74]](#endnote-72)^,^[[75]](#endnote-73)^ the polio vaccine^[[76]](#endnote-74)^ (a project that incidentally exploited crowdfunding *via* the March of Dimes) and more recently DNDi’s ASAQ/Coarsucam.^[[77]](#endnote-75)^

15.6 There are arguments, primarily from the current pharma industry, that patents are a necessary incentive for drug development, though the nature of the argument depends on a drug’s Net Present Value. There are arguments outside the pharma sector suggesting that patents shelter companies from the need to innovate,^[[78]](#endnote-76)^ and that financial return can be achieved through less monopolistic methods. A recurrent accusation is that prices of medicines are not linked to R&D costs but are instead set by what society (or more precisely health insurance systems) are willing to pay; the result of that argument is to suggest that the real cost of pharma R&D (recently formulated as $2.6B^[[79]](#endnote-77)^) is significantly less than often quoted.^[[80]](#endnote-78)^ The cost of developing a new chemical entity for sleeping sickness has been estimated as €40-130M.^[[81]](#endnote-79)^^[[82]](#endnote-80)^ Open research practices have the potential to reduce costs through efficiency savings derived from reduced duplication of effort (an open drug may fail, but it would fail only once), reduced transactional costs (principally simplified legal arrangements) and, in high-priority areas in-kind resources and public infrastructure may be brought to bear. If such changes do not reduce the actual cost of discovery, they can reduce the financing needs that are acknowledged as being a substantial component of the current cost of drug development.

**Outline Organisational Structure**

16.0 We would propose an umbrella organization of minimal size and physical resources as per other models of “virtual” pharma, *i.e.,* employing a CEO, and potentially intellectual drivers in priority disease areas. Any employees of such an organization cannot perform any tasks that are equivalent to unremunerated tasks being performed by the community.

16.1 The organization manages people at the top level of all projects, either *via* direct employment or through partnering on costs with granting agencies.

16.2 A director is responsible for an overall project (*e.g.,* a chemical series) with the remit to marshal experimental efforts and to overcome roadblocks, while upholding absolute standards of openness and data sharing. For example, the creation and management of both push and pull mechanisms (*e.g.,* attracting or investing funds for small prizes and the terms under which money would be released) or pursuing advance market commitments or license exclusivity to influence the terms of any project investment. Project directors could be “installed” by an agency (*e.g.,* a PDP) interested in applying the open source model in a specific area for a discrete period.

16.3 The projects would have associated advisors from other agencies and pharma, providing guidance and oversight.

16.4 For such a proposition to be viable requires i) the organization to administer multiple projects (to ensure mutual learning through shared experience), and ii) a disease pipeline to be populated artificially on day 1 by the conversion of existing closed projects outside the organization to open projects managed by the organization positioned at various stages of drug discovery, in order that there may be demonstrable outputs in a meaningful timeframe. This second requirement represents the initial challenge: the identification and conversion of flagship projects suitable for such a conversion (perhaps through purchase) from closed to open.

16.5 The funding of the organization could arise from:

a) Combinations of existing sources, described above, better leveraged by the promise of efficiency gains through openness.

b) A company (not-for-profit, or for-profit) whose income stream is a parallel industry (*e.g.,* open source software development, data services and consultancy for other organizations/sectors) and which may direct funding to the drug development organization by operating as a foundation. An analogous company in software is Red Hat, a company with a revenue stream based on open source software as a service, and which reinvests money in research.

16.6 The above alterations to the financing of therapeutic discovery and development are significant and without precedent. Major alterations to the financing of industries are possible where there are seen to be potential cost savings or efficiency gains, as exemplified by the radical alterations made in the last decade in the creation of the open access publishing industry, where there has been fundamental and sustainable change to a major funding model *via* the coordinated support of international funding and governmental agencies motivated in part by i) a desire for improved access to products and ii) a clear social good.

16.7 The license applicable to all research outputs would be CC-BY or equivalent, meaning any output may be used for any purpose, including the generation of revenue, provided the source is acknowledged. It would thus remain possible for project content to be taken by closed groups for local/commercial benefit, in much the same way as much of the highly profitable genomics industry is built upon the data derived from the human genome project.^[[83]](#endnote-81)^ It is unlikely downstream commercial activity would negatively impact the upstream source project since a) any closely allied intellectual property taken would likely be seen as both uninventive and restricting a common good, and b) desirable scientific/health aims are likely to be achieved faster by working with the larger open group (which has institutional momentum) rather than competing with it towards identical goals. One can envisage derived commercial activities that contribute back to the open source core through non-contracted goodwill payments. (Examples exist of contracted payments arising from retroactive charges where the products from free software are commercialized.^[[84]](#endnote-82)^) “Viral” licenses used in software such as the General Public License, requiring participants to license their own contributions in the same way as the parent project, are likely too restrictive and may deter contributors. CC-BY licenses are compatible with embargo periods on the release of contributions to the public (and therefore “trade secrets”).^36^ Such mechanisms would be strongly discouraged but cannot realistically be prevented. While they permit the possible financial gain to a contributor from exploiting a result derived from the project, they present the community at large with a quandary – has the work proposed by one individual already been carried out by another?

**Conclusion**

Through the development of various initiatives, and the realization that the current R&D model is both inefficient and unsustainable, we feel the time has arrived where we try something different, where the difference is a radical level of openness and a clear focus on true health needs. The scientific and social arguments are compelling for an open source approach to new pharmaceuticals. We describe above a point-by-point method by which the classical drug discovery and development process may be fragmented and reconstructed from open components that can inter-operate, just as a substantial work of art (a mosaic) may be constructed by the contributions of many, when those contributors can see and understand all the project details. The approach is inclusive, and therefore requires participants from the whole academic/industrial/public range, rather than setting up unproductive “us *vs.* them” structures. It is essential that everyone should benefit if there is to be the level of community buy-in necessary for such an unprecedented venture.

**Acknowledgements**

We would like to thank the Rockefeller Foundation for hosting a meeting^[[85]](#endnote-83)^ that acted as the stimulus for this article, and Open Society Foundations for support of that meeting, the delegates of which are thanked for stimulating discussions.

**Disclaimer**

The authors alone are responsible for the views expressed in this publication, which do not necessarily represent the decisions, policy, or views of their employing organizations.

1. Research and Development to Meet Health Needs in Developing Countries: Strengthening Global Financing and Coordination, Report of the Consultative Expert Working Group on Research and Development: Financing and Coordination, World Health Organization, **2012**, ISBN: 978 92 4 150345 7, <http://www.who.int/phi/cewg_report/en/> (retrieved Feb 17^th^ 2017) [↑](#endnote-ref-1)
2. R. Kneller, *Nat. Rev. Drug Disc.* **2010**, *9*, 867–882. DOI: 10.1038/nrd3251 [↑](#endnote-ref-2)
3. M. Abou-Gharbia and W. E. Childers, *J. Med. Chem*. **2014**, *57*, 5525–5553. DOI: 10.1021/jm401564r [↑](#endnote-ref-3)
4. M. Abou-Gharbia and W. E. Childers, *J. Med. Chem*. **2013**, *56*, 5659–5672. DOI: 10.1021/jm400330j [↑](#endnote-ref-4)
5. Address by WHO Director General Margaret Chan, Regional Committee for Africa, 64^th^ Session, Cotonou, Republic of Benin, 3^rd^ Nov 2014. <http://www.who.int/dg/speeches/2014/regional-committee-africa/en/> (retrieved Feb 17^th^ 2017) [↑](#endnote-ref-5)
6. Devalued and Distrusted: Can the Pharmaceutical Industry Restore its Broken Image, J. L. LaMattina, **2013**, Wiley. ISBN 978-1118487471 [↑](#endnote-ref-6)
7. M. Kessel, *Nat. Biotech*. **2014**, *32*, 983–990. DOI: 10.1038/nbt.3036 [↑](#endnote-ref-7)
8. D. W. Light and J. R. Lexchin, *BMJ* **2012**, *345*, e4348. DOI: 10.1136/bmj.e4348 [↑](#endnote-ref-8)
9. C. J. Thomas and J. C. McKew, *Curr. Top. Med. Chem.* **2014**, *14*, 291–293. DOI: 10.2174/1568026613666131127125351 [↑](#endnote-ref-9)
10. For simplicity in this article we will conflate new chemical entities and regimens. The strategies described herein are applicable to, but more general than, important current efforts in drug repurposing, which will typically save time and resources pre-Phase II in both open and closed development; see a) N. Novac, *Trends Pharmacol. Sci.* **2013**, *34*, 267–272 (DOI: 10.1016/j.tips.2013.03.004); b) A. Mullard, *Nat. Rev. Drug. Disc*. **2012**, *11*, 505–506 (DOI: 10.1038/nrd3776) [↑](#footnote-ref-1)
11. M. Goldman, C. Compton and B. B. Mittleman, *Clin. Trans. Med*. **2013**, *2*:2. DOI: 10.1186/2001-1326-2-2 [↑](#endnote-ref-10)
12. S. P. Forster, J. Stegmaier, R. Spycher and S. Seeger, *Drug Discovery Today* **2014**, *19*, 348–355. DOI: 10.1016/j.drudis.2013.11.015 [↑](#endnote-ref-11)
13. R. Atun, F. M. Knaul, Y. Akachi and J. Frenk, *Lancet* **2012**, *380*, 2044–2049. DOI: 10.1016/S0140-6736(12)61460-3 [↑](#endnote-ref-12)
14. D. Holmes, *Nature Med.* **2014**, *20*, 320. DOI: 10.1038/nm0414-320 [↑](#endnote-ref-13)
15. Prizes to Stimulate Innovation, Knowledge Ecology International, <http://www.keionline.org/prizes> (retrieved Feb 17^th^ 2017) [↑](#endnote-ref-14)
16. About UNITAID, <http://www.unitaid.eu/en/who/about-unitaid> (retrieved Feb 17^th^ 2017) [↑](#endnote-ref-15)
17. Strong Medicine: Creating Incentives for Pharmaceutical Research on Neglected Diseases; Michael Kremer & Rachel Glennerster; Princeton University Press, **2004**, ISBN 9780691121130 [↑](#endnote-ref-16)
18. The Pneumococcal Vaccine AMC, GAVI, <http://www.gavi.org/funding/pneumococcal-amc/> (retrieved Feb 17^th^ 2017) [↑](#endnote-ref-17)
19. T. Cernuschi, E. Furrer, N. Schwalbe, A. Jones, E. R. Berndt and S. McAdams, *Bull. World Health Org.* **2011**, *89*, 913–918. DOI: 10.2471/BLT.11.087700 [↑](#endnote-ref-18)
20. The AgResults Initiative: http://agresults.org/en/268/VisionMissionValues (retrieved Feb 17^th^ 2017) [↑](#endnote-ref-19)
21. D. B. Ridley, H. G. Grabowski and J. L. Moe, *Health Aff.* **2006**, *25*, 313–324. DOI: 10.1377/hlthaff.25.2.313 [↑](#endnote-ref-20)
22. A. C. Sanchez, *Pharmaceuticals Policy and Law* **2014**, *16*, 167–172. DOI: 10.3233/PPL-140374 [↑](#endnote-ref-21)
23. P. Doshi, *BMJ* **2014**, *349*:g4665. DOI: 10.1136/bmj.g4665 [↑](#endnote-ref-22)
24. A. Banerjee, A. Hollis and T. Pogge, *Lancet* **2010**, *375*, 166–169. DOI: 10.1016/S0140-6736(09)61296-4 [↑](#endnote-ref-23)
25. D. E. Fagnan, N. N. Yan, J. C. McKew and A. W. Lo, *Sci. Transl. Med*. **2015**, *7*, 276ps3. DOI: 10.1126/scitranslmed.aaa2360 [↑](#endnote-ref-24)
26. S. Frantz, *Nat. Rev. Drug Disc*. **2012**, *11*, 5. DOI: 10.1038/nrd3642 [↑](#endnote-ref-25)
27. The Medicines Patent Pool, <http://www.medicinespatentpool.org/> (retrieved Feb 17^th^ 2017) [↑](#endnote-ref-26)
28. World Intellectual Property Organization (WIPO) Re:Search, <http://www.wipo.int/research/en/> (retrieved Feb 17^th^ 2017) [↑](#endnote-ref-27)
29. Médecins Sans Frontières Access Campaign, https://www.msfaccess.org/spotlight-on/3p-project-new-approach-developing-better-treatments-tb (retrieved Feb 17^th^ 2017) [↑](#endnote-ref-28)
30. A. Mullard, *Nat. Rev. Drug Disc*. **2011**, *10*, 643–644. DOI: 10.1038/nrd3545 [↑](#endnote-ref-29)
31. C. G. Begley and L. M. Ellis, *Nature* **2012**, *483*, 531–533. DOI: 10.1038/483531a [↑](#endnote-ref-30)
32. S. M. Maurer, A. Rai and A. Sali, *PLOS Med*. **2004**, *1(3)*: e56. DOI: 10.1371/journal.pmed.0010056 [↑](#endnote-ref-31)
33. T. B. Kepler, M. A. Marti-Renom, S. M. Maurer, A. K. Rai, G. Taylor and M. H. Todd, *Aus. J. Chem*. **2006**, *59*, 291–294. DOI: 10.1071/CH06095 [↑](#endnote-ref-32)
34. S. M. Maurer, *Future Med. Chem*. **2013**, *5*, 895–906. DOI: 10.4155/fmc.13.65 [↑](#endnote-ref-33)
35. The Human Genome Project, <http://www.genome.gov/10001772> (retrieved Feb 17^th^ 2017) [↑](#endnote-ref-34)
36. The International HapMap Project, *Nature* **2003**, *426*, 789–796. DOI: 10.1038/nature02168 [↑](#endnote-ref-35)
37. PubChem, https://pubchem.ncbi.nlm.nih.gov/ (retrieved Feb 17^th^ 2017) [↑](#endnote-ref-36)
38. W. C. van Voorhis, J. H. Adams, R. Adelfio, V. Ahyong, M. H. Akabas, P. Alano, *et al.* *PLoS Pathog*. **2016**, *12*, e1005763. DOI: 10.1371/journal.ppat.1005763 [↑](#endnote-ref-37)
39. Structural Genomics Consortium Chemical Probes, <http://www.thesgc.org/chemical-probes> (retrieved Feb 17^th^ 2017) [↑](#endnote-ref-38)
40. Z. Arshad, J. Smith, M. Roberts, W. H. Lee, B. Davies, K. Bure *et al. Expert Opin. Drug Disc*. **2016**, *11*, 321–332. DOI: 10.1517/17460441.2016.1144587 [↑](#endnote-ref-39)
41. Open Source Drug Discovery, <http://www.osdd.net/> (retrieved Feb 17^th^ 2017) [↑](#endnote-ref-40)
42. A. E. Williamson, P. M. Ylioja, M. N. Robertson, Y. Antonova-Koch, V. Avery, J. B. Baell *et al. ACS Cent Sci* **2016**, *2*, 687–701. DOI: 10.1021/acscentsci.6b00086 [↑](#endnote-ref-41)
43. The Sysborg Platform, <http://www.osdd.net/about-us/how-osdd-works/sysborg-2-0> (retrieved Feb 17^th^ 2017) [↑](#endnote-ref-42)
44. M. N. Robertson, P. M. Ylioja, A. E. Williamson, M. Woelfle, M. Robins, K. A. Badiola, P. Willis, P. Olliaro, T. N. C. Wells and M. H. Todd, *Parasitology* **2014**, *141*, 148–157. DOI: 10.1017/S0031182013001121 [↑](#endnote-ref-43)
45. A. Bhardwaj, D. Bhartiya, N. Kumar, OSDD Consortium, and V. Scaria, *Tuberculosis* **2009**, *89*, 386–387. DOI: 10.1016/j.tube.2009.07.005 [↑](#endnote-ref-44)
46. The Synaptic Leap, 25^th^ July 2011, <http://www.thesynapticleap.org/node/343> (retrieved Feb 17^th^ 2017) [↑](#endnote-ref-45)
47. The Structural Genomics Consortium, <http://www.thesgc.org/> (retrieved Feb 17^th^ 2017) [↑](#endnote-ref-46)
48. Sage Bionetworks, http://sagebase.org/who-we-are/overview/ (retrieved Feb 17^th^ 2017) [↑](#endnote-ref-47)
49. In *open source* research all data and ideas are freely shared. *Crowdsourcing* involves a large number of people contributing to the solution of a problem, but the term does not necessarily imply that all the scientific details are shared. An example is the FoldIt project in which contributors solved an important problem in science through crowdsourcing, but were not involved in the generation of a resulting scientific model, see S. Cooper, F. Khatib, A. Treuille, J. Barbero, J. Lee, M. Beenen, A. Leaver-Fay, D. Baker, Z. Popovic and F. Players, *Nature* **2010**, *466*, 756–760 (10.1038/nature09304) [↑](#footnote-ref-2)
50. B. Y. Lee and D. S. Burke, *Vaccine* **2010**, *28*, 2806–2809. DOI: 10.1016/j.vaccine.2009.09.047 [↑](#endnote-ref-48)
51. W. Zheng, N. Thorne and J. C. McKew, *Drug Discovery Today* **2013**, *18*, 1067–1073. DOI: 10.1016/j.drudis.2013.07.001 [↑](#endnote-ref-49)
52. Crowdsourcing Drug Discovery, Intermolecular Blog, <http://intermolecular.wordpress.com/2014/10/03/crowdsourcing-drug-discovery/> (retrieved Feb 17^th^ 2017) [↑](#endnote-ref-50)
53. Research Experiences for College Students, IndiaBioscience, https://indiabioscience.org/columns/education/research-experiences-for-college-students (retrieved Feb 17^th^ 2017) [↑](#endnote-ref-51)
54. W. L. Scott and M. J. O’Donnell, *J. Comb. Chem*. **2009**, *11*, 3–13. DOI: 10.1021/cc800183m [↑](#endnote-ref-52)
55. SageBase Synapse, <https://www.synapse.org/> (retrieved Feb 17^th^ 2017) [↑](#endnote-ref-53)
56. S. L. Cummings and R. L. Sandefur, *Harvard Law & Policy Review* **2013**, *7*, 83–111. [↑](#endnote-ref-54)
57. N. Dragojlovic and L. D. Lynd, *Drug Discovery Today* **2014**, *19*, 1775–1780. DOI: 10.1016/j.drudis.2014.06.019 [↑](#endnote-ref-55)
58. Innocentive, https://www.innocentive.com/ (retrieved Feb 17^th^ 2017) [↑](#endnote-ref-56)
59. J. Bentzien, I. Muegge, B. Hamner and D. C. Thompson, *Drug Discovery Today* **2013**, *18*, 472–478. DOI: 10.1016/j.drudis.2013.01.002 [↑](#endnote-ref-57)
60. Matlab Competitions, https://au.mathworks.com/matlabcentral/contest/contests/3/statistics.html (retrieved Feb 17^th^ 2017) [↑](#endnote-ref-58)
61. Sage Bionetworks Dream Challenges, <http://dreamchallenges.org/> (retrieved Feb 17^th^ 2017) [↑](#endnote-ref-59)
62. Future Perfect: The Case for Progress in a Networked Age, Steven Johnson, **2012**, Riverhead, ISBN 978-1594488207 [↑](#endnote-ref-60)
63. For an outline of this idea, see “Molecular Craigslist”, Intermolecular Blog, **2012**, <http://intermolecular.wordpress.com/2012/04/20/molecular-craigslist/> (retrieved Feb 17^th^ 2017) [↑](#endnote-ref-61)
64. M. C. Carrillo *et al., Alzheimers Dement*. **2013**, *9*, 137–140. DOI: 10.1016/j.jalz.2012.11.003 [↑](#endnote-ref-62)
65. WHO/TDR Call for Expression of Interest: <http://www.who.int/tdr/grants/CL_Call_for_Expression_of_Interest.pdf> (retrieved Feb 17^th^ 2017) [↑](#endnote-ref-63)
66. V. S. Moorthy, G. Karam, K. S. Vannice, M.-P. Kieny *PLoS Med.* **2015**, *12*, e1001819. DOI: 10.1371/journal.pmed.1001819 [↑](#endnote-ref-64)
67. The Alltrials Campaign, <http://www.alltrials.net/> (retrieved Feb 17^th^ 2017) [↑](#endnote-ref-65)
68. A. Leiter, T. Sablinski, M. Diefenbach, M. Foster, A. Greenberg, J. Holland, W. K. Oh and M. D. Galsky, *J. Natl. Cancer Inst*. **2014**, *106*(10): dju258. DOI: 10.1093/jnci/dju258 [↑](#endnote-ref-66)
69. T. Sablinski, *Sci. Transl. Med*. **2014**, *6*, 256ed19. DOI: 10.1126/scitranslmed.3009116 [↑](#endnote-ref-67)
70. W. A. Kaplan, V. J. Wirtz and P. Stephens, *PLOS ONE* **2013**, *8(9)*: e74399. DOI: 10.1371/journal.pone.0074399 [↑](#endnote-ref-68)
71. J. Chakma, G. H. Sun, J. D. Steinberg, S. M. Sammut and R. Jagsi, *N. Engl. J. Med*. **2014**, *370*: 3–6. DOI: 10.1056/NEJMp1311068 [↑](#endnote-ref-69)
72. *Prescrire International* **2015**, *24*, 107–110, <http://english.prescrire.org/en/SummaryDetail.aspx?Issueid=159> (retrieved Feb 17^th^ 2017) [↑](#endnote-ref-70)
73. M. Woelfle, P. Olliaro and M. H. Todd, *Nature Chemistry* **2011**, *3*, 745–748. DOI: 10.1038/nchem.1149 [↑](#endnote-ref-71)
74. L. Palombi, *SCRIPTed* **2009**, *6:2*, 394 (<https://script-ed.org/wp-content/uploads/2016/07/6-2-Palombi.pdf>, accessed Feb 17^th^ 2017) [↑](#endnote-ref-72)
75. For more discussion on this history, see <http://archive.mises.org/5216/patent-and-penicillin/> (retrieved Feb 17^th^ 2017) [↑](#endnote-ref-73)
76. Patenting the Sun: Polio and the Salk Vaccine, J. Smith, **1991**, New York: Anchor/Doubleday. ISBN: 978-0688094942 [↑](#endnote-ref-74)
77. Low-Cost Antimalarial Pill Available, New York Times, March 1^st^ 2007, <http://www.nytimes.com/2007/03/01/health/01malaria.html> (retrieved Feb 17^th^ 2017) [↑](#endnote-ref-75)
78. M. Boldrin and D. K. Levine, *J. Econ. Perspect*. **2013**, *27*, 3–22. DOI: 10.1257/jep.27.1.3 [↑](#endnote-ref-76)
79. J. A. DiMasi, H. G. Grabowski and R. W. Hansen, Innovation in the Pharmaceutical Industry: New Estimated of R&D Costs, **2014**, Tufts Center for the Study of Drug Development [↑](#endnote-ref-77)
80. The Price of Failure, Economist, November 29^th^ 2014, <http://www.economist.com/news/business/21635005-startling-new-cost-estimate-new-medicines-met-scepticism-price-failure> (retrieved Feb 17^th^ 2017) [↑](#endnote-ref-78)
81. An Innovative Approach to R&D for Neglected Patients. Ten Years of Experience & Lessons Learned by DNDi, Drugs for Neglected Diseases Initiative, 2014, http://www.dndi.org/wp-content/uploads/2009/03/DNDi_Modelpaper_2013.pdf (retrieved Feb 17^th^ 2017) [↑](#endnote-ref-79)
82. Busting the billion-dollar myth: how to slash the cost of drug development, *Nature* **2016**, *536*, 388–390. DOI: 10.1038/536388a [↑](#endnote-ref-80)
83. Economic Return from Human Genome Project Grows, M. Wadman, *Nature* 12^th^ June **2013**. DOI: 10.1038/nature.2013.13187 [↑](#endnote-ref-81)
84. Free Public Domain Research License, OpenEye Scientific Software, <http://eyesopen.com/academic> (retrieved Feb 17^th^ 2017) [↑](#endnote-ref-82)
85. Open source pharma meeting discusses future, World Health Organisation, August 2014, <http://www.who.int/tdr/news/2014/open-source-pharma-mtg/en/> (retrieved Feb 17^th^ 2017) [↑](#endnote-ref-83)
